# Gallstone Ileus Presenting as Small Bowel Obstruction in an Elderly Patient: Diagnostic Approach and Surgical Management

**DOI:** 10.3390/jcm15208122

**Published:** 2026-10-19

**Authors:** Yerkhanat Khuanbai, Berik Rovgaliyev, Tlek Karimov, Ruslan Khassenov, Saken Kozhakhmetov

**Affiliations:** 1 Professor G.V. Tsoi Scientific and Educational Center for Surgery, Astana Medical University, Astana 010000, Kazakhstan; 2 Department of General Surgery, Multidisciplinary City Hospital №2, Astana 010000, Kazakhstan

**Keywords:** gallstone ileus, small bowel obstruction, cholecystoduodenal fistula, elderly patient, case report

## Abstract

**Background**: Gallstone ileus is an uncommon cause of mechanical intestinal obstruction that predominantly affects older adults. It results from migration of a gallstone through a biliary–enteric fistula, most commonly a cholecystoduodenal fistula. **Methods**: An 84-year-old woman with hypertension and hypothyroidism presented with a four-day history of right upper quadrant abdominal pain, nausea, and vomiting. Laboratory investigations showed a marked inflammatory response and severe renal dysfunction with a substantial reversible acute component, most likely related to volume depletion and reduced renal perfusion. Computed tomography demonstrated an ectopic gallstone impacted in the distal small bowel, proximal bowel dilatation, pneumobilia, and a cholecystoduodenal fistula. Emergency laparotomy confirmed gallstone ileus. After assessment of the operative risk and local anatomy, a one-stage procedure was performed, comprising enterolithotomy, cholecystectomy, division of the fistula, and primary repair of the duodenal defect. **Results:** Postoperative management included continued monitoring of the admission renal dysfunction, together with treatment of postoperative tracheobronchitis, anemia, electrolyte disturbances, and transient elevation in pancreatic enzymes. These abnormalities were managed with supportive and multidisciplinary care. Renal function, inflammatory markers, and electrolyte concentrations improved substantially before discharge. Pancreatic amylase returned to its reference interval, whereas total α-amylase was below its stated reference interval at discharge, although anemia persisted. The patient was discharged in satisfactory condition on postoperative day 13. **Conclusions**: Computed tomography enabled preoperative diagnosis and characterization of the cholecystoduodenal fistula. Although a one-stage operation was feasible in this patient, this single outcome does not establish its superiority. Surgical management should be individualized according to the patient’s physiological condition, comorbidities, and intraoperative findings.

## 1. Introduction

Gallstone ileus is an uncommon form of mechanical intestinal obstruction caused by migration and impaction of a gallstone within the gastrointestinal tract [1]. It occurs in approximately 0.3–0.5% of patients with gallstones and accounts for approximately 1–4% of mechanical intestinal obstructions, with a disproportionate burden among older adults [2,3]. Women are affected more frequently than men [3]. The condition usually develops when chronic gallbladder inflammation and pressure necrosis lead to the formation of a biliary–enteric fistula, most commonly a cholecystoduodenal fistula [3,4]. A sufficiently large stone may then enter the intestinal lumen and become impacted, usually in the terminal ileum or near the ileocecal valve [1]. Clinical manifestations, including intermittent abdominal pain, nausea, vomiting, abdominal distension, and constipation, are often nonspecific. Marked signs of peritoneal irritation may be absent, which can contribute to delayed diagnosis [5]. Computed tomography is the imaging modality of choice because it can demonstrate the components of Rigler’s triad, namely intestinal obstruction, an ectopic gallstone, and pneumobilia. It may also directly demonstrate the biliary–enteric fistula [6]. Surgical treatment is generally required, but the optimal extent of surgery remains controversial. The principal options are enterolithotomy alone, a one-stage procedure combining enterolithotomy with cholecystectomy and fistula repair, and a two-stage procedure involving initial enterolithotomy followed by delayed biliary surgery. Selection of the surgical approach depends on the patient’s physiological condition, comorbidities, intraoperative anatomy, and the anticipated risks associated with a more extensive operation [3,7]. We report the case of an 84-year-old woman with gallstone ileus caused by a cholecystoduodenal fistula who underwent a one-stage operation. This case illustrates the diagnostic contribution of computed tomography, the factors considered when selecting the surgical approach, and the importance of careful postoperative management in an older patient with substantial comorbidity.

## 2. Case Presentation

### 2.1. Clinical History and Presentation

An 84-year-old woman presented to the emergency department with a four-day history of nausea, vomiting, and progressively worsening right upper quadrant abdominal pain. Her medical history included hypertension treated with regular antihypertensive medication and hypothyroidism. No reliable pre-admission creatinine value was available, and no confirmed stage of chronic kidney disease was documented. She had no history of previous surgery and no known allergies.

### 2.2. Physical Examination Findings

On admission, the patient was conscious, fully oriented, and in a moderately ill general condition. She was afebrile, with a body temperature of 36.5 °C. Her blood pressure was 130/80 mmHg, and her heart rate was 75 beats/min. She was hemodynamically stable. Cardiovascular, respiratory, and neurological examinations revealed no clinically significant abnormalities.

The abdomen was mildly distended but remained soft on palpation. Mild diffuse tenderness was present, predominantly in the upper and central abdomen. There was no guarding, rebound tenderness, or other evidence of peritoneal irritation. Bowel sounds were increased and intermittently high-pitched. Passage of flatus and stool was reduced. No abdominal masses or external hernias were identified. These findings, together with the history of nausea and vomiting, were suggestive of mechanical small-bowel obstruction.

### 2.3. Laboratory Investigations

Admission laboratory tests showed a marked inflammatory response, with a C-reactive protein level of 108.8 mg/L. Renal function was severely impaired, with a serum creatinine concentration of 440.97 µmol/L and a urea concentration of 36.45 mmol/L. In the context of four days of vomiting, reduced oral intake, and mechanical intestinal obstruction, the renal dysfunction was considered predominantly acute and most likely related to volume depletion and reduced renal perfusion. Creatinine decreased progressively after surgery and supportive treatment, reaching 73 µmol/L by discharge, which supported a substantial reversible component. However, because a reliable pre-admission creatinine value and quantified urine-output data were unavailable, the baseline renal function and formal stage of acute kidney injury could not be determined retrospectively. Alanine aminotransferase was mildly elevated at 54.61 U/L, while total and direct bilirubin levels were within the reference ranges. Alpha-amylase was mildly elevated at 116.57 U/L. Renal function and inflammatory markers improved following surgery and supportive treatment. Serial laboratory findings are summarized in Table 1.

### 2.4. Imaging Findings

**Figure 1:**
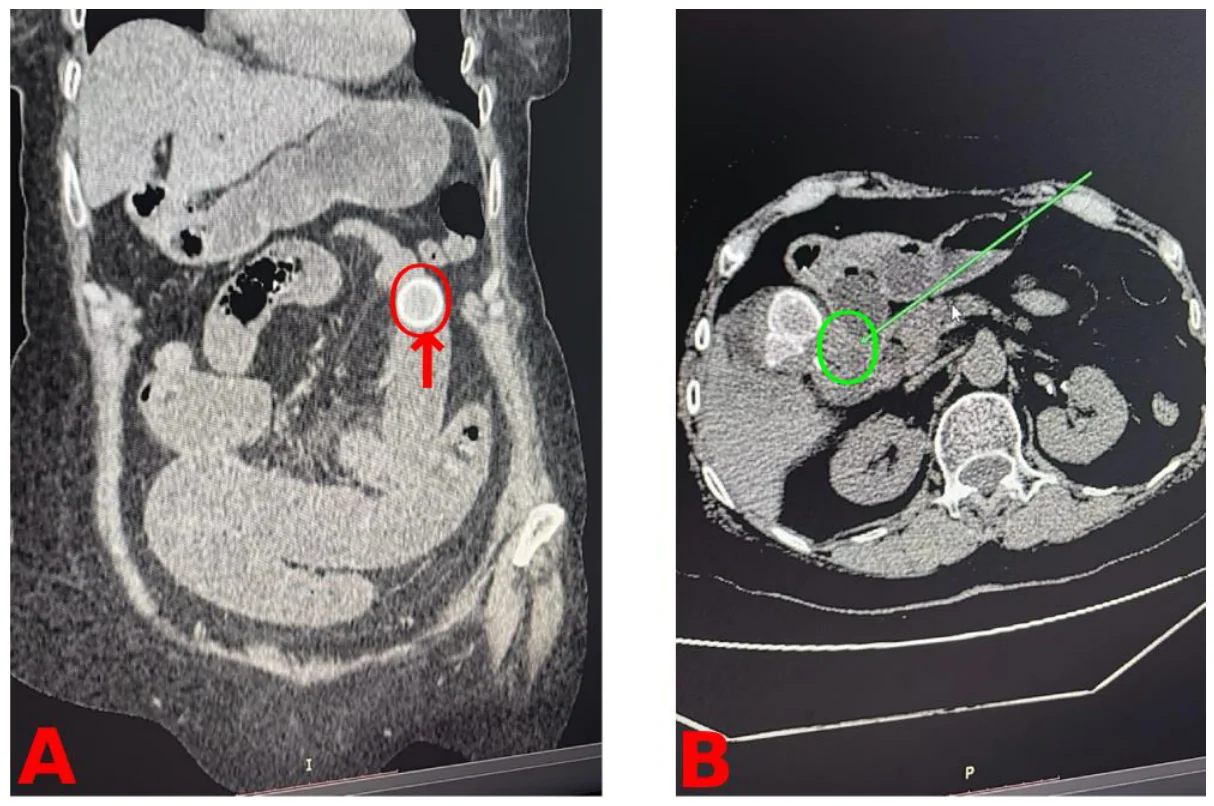
Preoperative CT Imaging of Gallstone Ileus. (**A**) Coronal CT image demonstrating an ectopic gallstone impacted in the distal small bowel (red arrow and circle), with proximal small-bowel dilatation. (**B**) Axial CT image demonstrating the cholecystoduodenal fistula connecting the gallbladder and duodenal bulb, indicated by the radiologist’s green arrow and circle.

Abdominal ultrasonography demonstrated multiple gallstones, gallbladder wall thickening, and dilated small-bowel loops. Abdominal CT provided the principal diagnostic findings. Coronal images showed a calcified ectopic gallstone impacted in the distal small bowel, with proximal bowel dilatation and a transition point consistent with mechanical obstruction (Figure 1A). Axial images demonstrated pneumobilia and a cholecystoduodenal fistula connecting the gallbladder to the duodenal bulb, with mild surrounding inflammatory changes (Figure 1B). There was no CT evidence of bowel ischemia, perforation, or significant free intraperitoneal fluid.

Preoperative assessment showed preserved left ventricular systolic function, with an ejection fraction of 60% and no significant valvular or regional wall-motion abnormalities. Electrocardiography showed sinus rhythm, and chest radiography demonstrated chronic bronchitic changes without focal pulmonary consolidation. Lower-extremity Doppler ultrasonography showed bilateral atherosclerotic arterial changes without venous thrombosis.

The clinical and CT findings established the diagnosis of gallstone ileus caused by a cholecystoduodenal fistula. Urgent surgical treatment was undertaken to relieve the mechanical small-bowel obstruction.

### 2.5. Surgical Intervention

**Figure 2:**
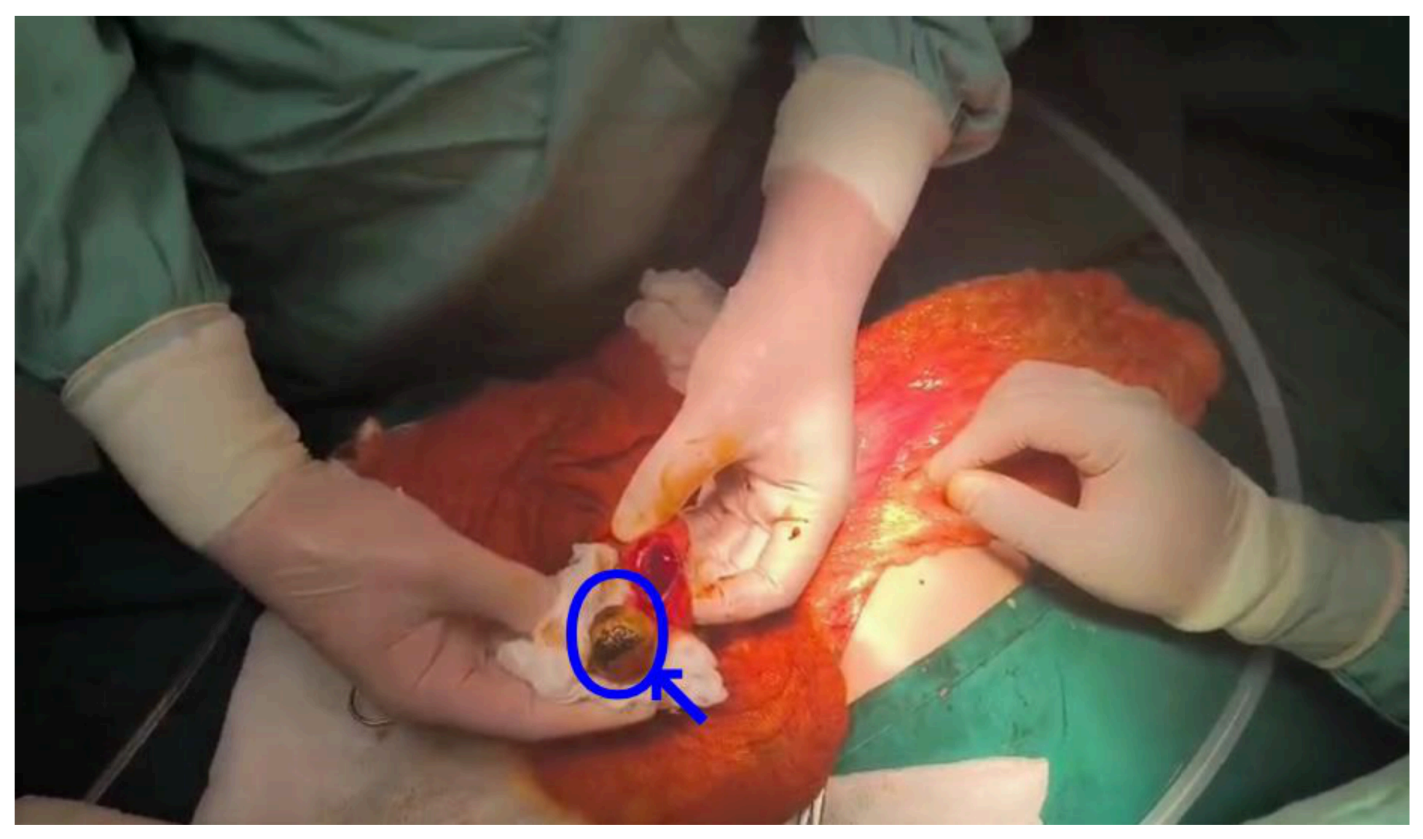
Intraoperative Findings. Intraoperative view showing the extracted gallstone (blue arrow and circle, approximately 2.5 cm in diameter).

Emergency exploratory laparotomy was performed for mechanical small-bowel obstruction caused by an ectopic gallstone. Dense adhesions were encountered in the right upper quadrant. A gallstone was impacted in the distal ileum, causing complete luminal obstruction and marked proximal bowel dilatation. A longitudinal enterotomy was made over the site of impaction, and a pigmented gallstone measuring approximately 2.5 cm was extracted (Figure 2). The enterotomy was then closed in two layers.

Further exploration revealed a chronically inflamed gallbladder that was densely adherent to the duodenal bulb, with a well-established cholecystoduodenal fistula. Although the patient’s advanced age and severe renal dysfunction with a substantial reversible acute component on admission increased the operative risk, several clinical and anatomical factors supported definitive treatment during the same operation. She was hemodynamically stable before surgery and echocardiography demonstrated preserved left ventricular systolic function. CT showed no evidence of bowel ischemia or perforation. Following enterolithotomy, the bowel was viable and did not require resection. In addition, the fistulous tract was clearly identified and considered technically accessible for division and primary repair. After balancing these factors against the risks associated with a more extensive procedure, the surgical team proceeded with cholecystectomy, division of the fistula, and primary two-layer closure of the duodenal defect using sutures. The abdominal cavity was irrigated, hemostasis was confirmed, and drains were placed in the subhepatic and pelvic spaces. The operative diagnosis was gallstone ileus causing acute mechanical small-bowel obstruction in association with cholelithiasis, chronic calculous cholecystitis, and a cholecystoduodenal fistula.

### 2.6. Postoperative Course

Following surgery, the patient was admitted to the intensive care unit for postoperative monitoring and supportive care. She initially required mechanical ventilation but was extubated later the same day and maintained adequate spontaneous respiration with supplemental oxygen. She remained hemodynamically stable without vasopressor support. On POD 1, she developed postoperative tracheobronchitis, which was treated with bronchoscopy, therapeutic lavage, and antimicrobial therapy. Her condition improved, and she was transferred to the surgical ward on POD 2.

Serial laboratory monitoring showed progressive improvement in renal function. Creatinine decreased from 440.97 µmol/L on admission to 73 µmol/L at discharge. This rapid improvement supported a substantial reversible component, most likely related to correction of volume depletion and relief of the intestinal obstruction. Sodium reached a maximum of 158 mmol/L, while potassium ranged from 3.1 to 3.3 mmol/L during recovery. These electrolyte abnormalities were managed with adjustment of fluid therapy and electrolyte replacement and normalized by discharge, when sodium was 139 mmol/L and potassium was 3.9 mmol/L.

Total α-amylase and pancreatic amylase increased transiently during the postoperative period, reaching 269 U/L and 244.8 U/L, respectively, on POD 9. No postoperative diagnosis of acute pancreatitis was documented. At discharge, pancreatic amylase was within its stated reference interval at 19.17 U/L, whereas total α-amylase was below its stated reference interval at 21.3 U/L. Therefore, these values were described as having decreased substantially rather than having both normalized. CRP decreased from a postoperative peak of 251.2 mg/L to 3.52 mg/L at discharge.

Hemoglobin decreased from 132 g/L on admission to 87 g/L on POD 3 and remained reduced at 92 g/L at discharge. The patient remained hemodynamically stable, and no clinically apparent active bleeding was documented. No blood transfusion was required. Prothrombin time and INR were transiently prolonged, reaching 18.7 s and 1.72 on POD 3, respectively, and returned to their reference intervals by discharge. No clinically apparent thrombotic complication or specific treatment directed at these residual coagulation abnormalities was documented. Continued clinical and laboratory monitoring after discharge was recommended. The abdominal drains were removed after their output decreased, and the patient was discharged in satisfactory condition on POD 13, with surgical and internal medicine follow-up arranged. Post-discharge clinical and laboratory follow-up data were not available at the time of case report preparation, which limited the assessment of longer-term outcomes.

## 3. Discussion

Gallstone ileus is an uncommon but serious complication of cholelithiasis, occurring in approximately 0.3–0.5% of patients with gallstones and accounting for approximately 1–4% of mechanical small-bowel obstructions [3,8]. It predominantly affects older adults, with reported mean ages of 70–80 years, and is more common in women [9]. Our patient was an 84-year-old woman with hypertension, hypothyroidism, and severe renal dysfunction on admission. Her advanced age and acute physiological impairment increased the diagnostic complexity and perioperative risk.

Gallstone ileus usually develops after chronic gallbladder inflammation causes adhesion between the gallbladder and an adjacent segment of the gastrointestinal tract. Persistent inflammation and pressure from a gallstone can produce tissue necrosis and eventually form a biliary–enteric fistula, most commonly a cholecystoduodenal fistula [10]. A large gallstone can then enter the gastrointestinal tract and migrate distally until it becomes impacted at a relatively narrow site, usually the terminal ileum or ileocecal valve [11]. This sequence was evident in our patient. CT demonstrated pneumobilia, a cholecystoduodenal fistula, an ectopic gallstone in the distal small bowel, and proximal bowel dilatation. At laparotomy, a pigmented gallstone measuring approximately 2.5 cm was found impacted in the distal ileum. A well-established fistula between the chronically inflamed gallbladder and the duodenal bulb was also identified. The clinical presentation of gallstone ileus is often nonspecific and may delay diagnosis [10,12]. Our patient presented with right upper quadrant abdominal pain, nausea, and vomiting. Although these symptoms were consistent with intestinal obstruction, they may also occur in several biliary and gastrointestinal disorders. Importantly, she had mild abdominal tenderness but no guarding, rebound tenderness, or other signs of peritoneal irritation. The intermittent movement of a gallstone before its final impaction, described as the “tumbling stone” phenomenon, may further contribute to fluctuating symptoms and delayed recognition [10]. This possibility should be considered when an older patient with gallstone disease presents with symptoms of bowel obstruction. Imaging is central to the diagnosis of gallstone ileus [13]. Ultrasonography is accessible and useful for identifying gallstones, gallbladder abnormalities, and dilated bowel loops, although its diagnostic performance may be limited by bowel gas and operator dependence [14]. In our patient, ultrasonography demonstrated multiple gallstones, gallbladder wall thickening, and dilated small-bowel loops. CT provided the decisive findings. It demonstrated all components of Rigler’s triad, consisting of intestinal obstruction, an ectopic gallstone, and pneumobilia, together with direct visualization of the cholecystoduodenal fistula. CT has a reported sensitivity of approximately 93% for gallstone ileus and provides useful information about the site of obstruction, fistula anatomy, and potential complications [13]. Although the complete Rigler’s triad is not present in every patient, its recognition strongly supports the diagnosis [13]. In this case, CT allowed the diagnosis to be established before surgery and showed no evidence of bowel ischemia or perforation.

The optimal surgical approach remains controversial because the available evidence is based mainly on retrospective studies and historical case series rather than randomized comparisons [8].

Enterolithotomy alone directly relieves the obstruction and generally requires a shorter and less extensive operation. It is therefore commonly preferred for frail, unstable, or severely comorbid patients. However, it leaves the fistula and any residual gallstones untreated, resulting in a reported recurrence risk of approximately 5–8% [8]. A one-stage procedure combines enterolithotomy with cholecystectomy and fistula repair. It provides definitive treatment but requires more extensive dissection and may prolong operative time. Reported differences in outcomes between one-stage surgery and enterolithotomy alone should be interpreted cautiously because of selection bias. More stable patients with favorable anatomy are more likely to undergo definitive surgery, whereas enterolithotomy alone is often selected for patients with greater physiological risk. One-stage surgery should therefore be reserved for carefully selected patients in whom the operative findings and physiological condition permit a more extensive procedure [15]. A two-stage strategy, with delayed biliary surgery after initial enterolithotomy, may be considered when definitive treatment is not appropriate during the emergency operation but persistent or recurrent biliary disease later requires intervention [16].

In this patient, the decision to perform a one-stage operation was individualized. Factors supporting definitive treatment included stable preoperative hemodynamics, preserved left ventricular systolic function, the absence of bowel ischemia or perforation, the absence of a need for bowel resection, and a clearly identifiable fistula that was considered technically accessible for division and primary closure. These factors were carefully weighed against the substantial operative risks associated with the patient’s advanced age and renal dysfunction on admission. The favorable outcome demonstrates that one-stage surgery was feasible in this individual patient, but it does not establish the superiority or general suitability of this approach in elderly or high-risk patients.

The postoperative course included continued recovery from the admission renal dysfunction, brief mechanical ventilation, postoperative tracheobronchitis, anemia, hypernatremia, hypokalemia, and transient elevation in pancreatic enzymes. The patient improved sufficiently for transfer from the ICU on POD 2 and was discharged on POD 13. These individual findings should be distinguished from population-level morbidity and mortality. Published outcomes vary because patients differ in age, comorbidities, physiological condition, and surgical management [17].

Renal function and electrolyte abnormalities improved with supportive care and fluid and electrolyte correction. The pancreatic enzyme elevations were transient. At discharge, pancreatic amylase had returned to its reference interval, whereas total α-amylase was below its stated reference interval. Because no postoperative diagnosis of acute pancreatitis was documented, enzyme elevation alone was not interpreted as evidence of acute pancreatitis. Anemia persisted at discharge, with a hemoglobin concentration of 92 g/L. No post-discharge clinical or laboratory data were available, which limits long term assessment. Coordinated care from the surgical, anesthesiology, intensive care, nephrology, cardiology, and internal medicine teams supported the management of the patient during hospitalization.

## 4. Conclusions

Gallstone ileus should be considered in older patients presenting with mechanical small-bowel obstruction, even when symptoms are nonspecific and signs of peritoneal irritation are absent. In this case, CT demonstrated the obstructing ectopic gallstone, pneumobilia, proximal bowel dilatation, and a cholecystoduodenal fistula, allowing the diagnosis to be established before surgery.

A one-stage procedure was selected after assessment of the operative risk and intraoperative anatomy. Although the patient recovered, this single outcome does not establish the superiority or general suitability of one-stage surgery in elderly or high-risk patients. Enterolithotomy alone remains an important option when the additional operative burden of cholecystectomy and fistula repair is considered unsafe. Surgical treatment should therefore be individualized according to the patient’s physiological condition, comorbidities, and operative findings. Further comparative evidence is required to define appropriate selection criteria for each surgical approach.

## Figures and Tables

**Table 1:** Serial laboratory findings from admission to discharge.

Parameter (Unit)	Reference Range	Admission Day	POD3	POD5	POD7	POD9	Discharge Day
**Inflammatory Markers**							
C-reactive protein (CRP), mg/L	<5	108.8	251.2	92.8	37.7	27.7	3.52
**Renal Function**							
Creatinine, µmol/L	44–80	440.97	162.96	125.96	119	93	73
Urea, mmol/L	2.5–7.5	36.45	31.27	26.28	19.4	11.1	4.0
**Liver Function**							
ALT, U/L	<40	54.61	43.99	50.32	29.5	23.2	15.1
AST, U/L	<40	35.19	81.26	60.14	32.2	32.8	27.0
Total bilirubin, µmol/L	<20	9.54	12.3	9.92	9	7.0	6.2
Direct bilirubin, µmol/L	<5	3.22	5.72	4.56	4.4	2.5	2.6
**Metabolic & Nutritional**							
Glucose, mmol/L	3.9–6.1	7.42	—	5.86	6.77	5.46	5.77
Albumin, g/L	35–50	—	33.71	35.67	37.9	35.3	32.0
Total protein, g/L	60–80	—	48.13	52.8	55.6	56.3	49.2
**Pancreatic Enzymes**							
Alpha-amylase, U/L	28–100	116.57	49.11	226.83	203	269	21.3
Pancreatic amylase, U/L	13–53	—	23.9	190.2	182.8	244.8	19.17
**Electrolytes**							
Sodium (Na^+^), mmol/L	136–145	—	152	157	158	148	139
Potassium (K^+^), mmol/L	3.5–5.0	—	3.8	3.1	3.3	3.3	3.9
**Complete Blood Count**							
Hemoglobin, g/L	120–160	132	87	91	81	87	92
White blood cells (WBC), ×10^9^/L	4.0–9.0	8.01	12.32	10.89	12.5	16.94	9.53
Platelets, ×10^9^/L	150–400	463	212	205	205	384	466
Hematocrit, %	35–45	40.7	27	28.8	27	29.5	28.3
Erythrocytes, ×10^12^/L	3.8–5.0	4.55	2.93	3.11	2.83	3.03	3.14
**Coagulation**							
Fibrinogen, g/L	1.8–4.0	5.78	5.02	5.13	3.55	3.17	3.35
APTT, sec	25–35	24.6	32	26.9	26.1	32.1	26.8
Prothrombin time, sec	10–15	11.5	18.7	17.2	17.9	13.5	12.5
INR	0.8–1.2	1.06	1.72	1.58	1.64	1.24	1.15
Thrombin time, sec	14–20	14.4	18.3	15.7	17.9	21.2	16.1
Antithrombin III, %	80–120	—	52.7	—	—	77.7	66.6
RFMC, mg%	<8	—	24	—	—	12.0	21.0

POD—postoperative day.

## Data Availability

The data presented in this study are available from the corresponding author upon reasonable request.
